# Co-application of proline or calcium and humic acid enhances productivity of salt stressed pomegranate by improving nutritional status and osmoregulation mechanisms

**DOI:** 10.1038/s41598-022-17824-6

**Published:** 2022-08-22

**Authors:** Ahmed AbdelHady Rashedy, Medhat Hamid Abd-ElNafea, Emad Hamdy Khedr

**Affiliations:** Pomology Department, Faculty of Agriculture, Cairo University, Giza, Egypt

**Keywords:** Ecology, Physiology, Plant sciences

## Abstract

Maximizing food production through integrated management of vegetative and root growth is a major challenge to food security and sustainability in the face of population growth, salinity stress conditions and climatic changes specially in arid and semi-arid regions. This study was conducted to evaluate the effect of foliar application with proline (Pro) at 5 mM, calcium (Ca) at 1.5% or control supplemented with soil application of humic acid (Hc) at 0, 15 g/tree on the nutrition status, osmoregulatory mechanisms and productivity of ‘Wonderful’ pomegranate trees growing under salt stress conditions. Soil and foliar treatments were applied three times: at flowering stage (April), 2 months after fruit set (June) and at fruit maturity (August). Individual application of either Hc or Pro or Ca alleviated the adverse effects of salt stress. Moreover, supplemented soil application of Hc with Pro or Ca as foliar application increased significantly leaf Pro, total carbohydrates, N, P, Ca and K contents, as well as K/Na and Ca/Na ratio. While it significantly decreased leaf Na and Cl concentration. Furthermore, supplemented application of Hc resulted in the highest decrease in leaf Na and Cl concentrations by 94.59%, 44.79% when combined with Pro and by 51.35%, 31.28%, when combined with Ca. In addition, Hc treatment led to the highest mean fruit yield by 139.56% and 90.73%, respectively as mean of both seasons for Pro and Ca treatments, respectively. The results suggest that, exogenous Pro and Ca supplemented with Hc can mitigate salt stress in ‘Wonderful’ pomegranate through enhancing osmoprotectants accumulaton.

## Introduction

Pomegranate (*Punica granatum* L.) consider one of the oldest edible fruits, mentioned in ancient Egyptian mythology and in the Holy Quran. It is widely grown in arid and semi-arid regions all over the world^1^. Pomegranate is more tolerant of dry and semi-arid regions for many reasons, such as adapting to hot, dry summers^1^, drought tolerance^2^ and moderate salt tolerance^3,4^. Consequently, pomegranate production has expanded worldwide, as the quality of available water decreases, such as salt water or recycled water^1^. In addition to the high nutritional value of pomegranate fruits, which have a wide range of medicinal applications^5^. ‘Wonderful’ is currently one of the most competitive pomegranate varieties grown in Egypt as it provides the optimum balance between productivity and quality^6^.

In arid and sem-arid regions, salinity as abiotic stress considers one of the most important environmental factors restricting agricultural productivity. Approximately, twenty percent of the irrigated land and two percent of the dry land agriculture have been affected by salinity worldwide^7^. Salinity resulted in reduced water use, plant metabolic processes changes, specific ions phytotoxicity (Na, Cl) and nutrition imbalance which reduces growth and productivity^8,9^.

Pomegranate is moderately salt tolerant species^4^. Cuttings of cv. ‘Malas Shirin’ pomegranate can tolerate up to 40 mM of NaCl in potted cultures^3^. Although, El-Khawaga et al.,^10^ reported that the pomegranate cvs. 'Wonderful’, ‘Manfalouty’ and ‘Nab-Elgamal’ pomegranates under a rate of 6.0 dS m^–1^ of groundwater had increased leaf Cl concentrations as well as reduced vegetative growth, flowering, and fruiting. On the other hand, pomegranate plants can limit Cl and Na accumulation in leaf tissues tolerating salinity up to EC of 15 dS m^−1^^4^.

Humic acid (Hc) helps plants mitigate harmful effects of soil salinity by improving soil permeability, aeration, holding capacity, particle aggregation and micronutrient uptake^11–13^. Also, humic acid reduces the uptake of toxic elements^8,13,14^.

Proline (Pro) accumulation in plants occur naturally under various stress conditions^8,15–17^. In addition, proline is involved in cytoplasmic osmotic adjustment, stabilization of proteins and membranes, buffering cellular redox potential and scavenging reactive oxygen species^15,18–20^. Moreover, the combined application of 10 mM ascorbic acid + 50 mM proline + 100 mM glycin betaine mitigated the negative effects of climatic change stresses in ‘Wonderful’ pomegranate trees^21^. More recently, exogenous proline reduced the uptake and translocation of Na and Cl^9,22^.

Calcium ions (Ca) are important for regulating the selective transport of K^+^ versus Na^+^ and maintaining cell membrane integrity^23–25^. Sodium ions act by displacing calcium ions from membranes, resulting in increased membrane permeability and elevated intracellular sodium^26,27^. Ca has very low mobility inside plant cells^28^.

There are little investigations on the effects of salinity on nutritional status, osmoregulation mechanism and productivity of pomegranate trees^4^. Also, the individual effects of calcium or Hc on stress tolerance have received much attention, but little information is available on the effects of the applications of Pro and Ca in combination with Hc on the yield and nutritional status of pomegranate trees. This study was conducted to evaluate the integrative effect of Pro or Ca as foliar application in addition to Hc as soil application on the yield and nutritional status of ‘Wonderful’ pomegranate trees under salt stress conditions.

## Material and methods

### Plant materials

The authors identify that the institutional and/or licensing committee that approved the experiments, including any relevant details, confirm that all experiments were performed in accordance with relevant named guidelines and regulations. This field study was conducted during 2019 and 2020 seasons along with 2021 season for laboratory analysis on 10-year-old pomegranate trees (*Punica granatum* L. cv. Wonderful) at the experimental station of Cairo University located in Wadi El Natrun, El Behera Governorate, Egypt (30° 41′ 42″ N and 30° 23′ 16″ E, altitude 9 m). Trees were planted 2.5 × 4 m apart in a sandy soil and were irrigated with saline water (Table 1).

**Table 1:** Chemical and physical characteristics of soil sample at the orchrd.

Sample	pH	EC dS/m	Soluble anions (meq^−l^)				Soluble cations (meq^−l^)		
			HCO_3_	Cl	SO_4_	Na	K	Ca	Mg
Soil	7.98	5.22	0.5	67.0	0.3	43.38	0.33	11.1	3.2
Water	7.35	4.1	3.8	27.2	14.58	35.1	0.48	6.0	4.0

Soil physical analysis (%)	Sand	Silt	Clay	Texture class	Organic matter (%)				
	94.15	4.35	1.5	Sandy	0.51				

### Treatments and procedures

Fifty-four trees were subjected to the common horticultural practices and sprayed with either chelated calcium citrate at 1.5%, proline at 0.5 g/L or water as control for foliar treatments using Tween 80 (0.5% v/v) as surfactant. Treated trees in every treatment divided into two groups; first group was treated with 15 g humic acid (Bio Green, Greensboro, GA, USA) as a soil application, and the other trees were not treated with humic acid. All foliar and soil treatments were applied during three critical stages; at flowering stage (April), 2 months after fruit set (June) and at fruit maturity (August). Each treatment consisted of nine trees distributed in three replicates, each one including 3 trees.

### Measurements and procedures leaf proline content (µmoles g^−1^)

The proline content was determined by the following procedure according to Bates et al.^29^. Leaf samples from each replicate were homogenized in 3% sulphosalicylic acid. After well shake, the samples were treated in a test tube with a mixture of glacial acetic acid and ninhydrin. Then the mixture was heated at 98 °C for 60 min in a water bath and then rapidly cooled at room temperature using crushed ice. Toluene was used to extract proline from the mixture and absorbance was read at 520 nm using spectrophotometer (6300 Visible spectrophotometer, Jenway, Cole-Parmer Ltd., United Kingdom).

### Leaf total carbohydrates

Total carbohydrates determination was carried out in the second season and was performed according to Herbert et al.^30^ as follows; a sample of dry tissue (0.2 g) was added in 10 ml H_2_SO_4_ (1 N). Then it was placed in a tube overnight in the oven at 100 °C. The colorimetric method used to determine total sugars^31^ was as follows: 1 ml of the sugar solution was mixed with 1 ml of 5% phenol followed by 5.0 ml of concentrated H_2_SO_4_ (98%). After shaking the tubes well, they were placed in a water bath at a temperature of 23–30 °C for 20 min. Then optical density of the color was measured at 490 nm using spectrophotometer.

### Determination of leaf mineral concentrations

Leaf samples were collected in the second season at August from the middle part of the shoots and they were dried at 70 °C for 72 h. Chloride determination was performed according to Mastrogiannidou et al.^32^ after extracting Cl with distilled water from dried tissues of leaves and titrated with silver nitrate solution (0.02 N). For Na, Ca, and K analyses, 0.2 g of the dried sample was digested into a mixture of H_2_SO_4_ and H_2_O_2_^33^. The concentrations of Na, Ca, and K were determined using a flame photometer (PFP7, Jenway, Cole-Parmer Ltd United Kingdom) according to Temminghoff and Houba^33^. Nitrogen concentration was determined using the modified Micro-Kjeldahl method^34^. Phosphorus concentration was determined sepectrophotometerically by using stannous chloride method^35^. All mineral concentrations are expressed as percent on a dry weight basis.

### Yield and fruit characteristics

The fruits were harvested at the maturity stage during the end of August (180 days after flowering). The fruit yield was calculated by multiplying the weight of the fruits by the number of fruits per tree. Fruit characteristics were determined using nine fruits per each replicate. Fruit weight was determined using a sensitive scale, fruit firmness was measured on the two opposite sides of each fruit (using penetrometer with an 8 mm diameter probe, FT 327). The fruit pulp and peel were weighed separately for the same fruit and then their ratio were calculated. A digital hand refractometer (PR32, ATago, CO. Ltd., Japan) was used to determine the total soluble solids (TSS) in the extracted juice and was expressed as ^◦^Brix.

### Experiment design and statistical analysis

The experiment design was a split plot with soil application in the main plot and the foliar application in the sub plot. The treatments were arranged in a randomized complete block design with two factors (2 soil treatments X 3 foliar treatments) and subjected to variance analysis^36^. The means of the treatments were compared by least significant difference at 0.05 significance level.

## Results and discussion

### Proline and carbohydrates as organic osmolytes for osmoregulation

**Figure 1:**
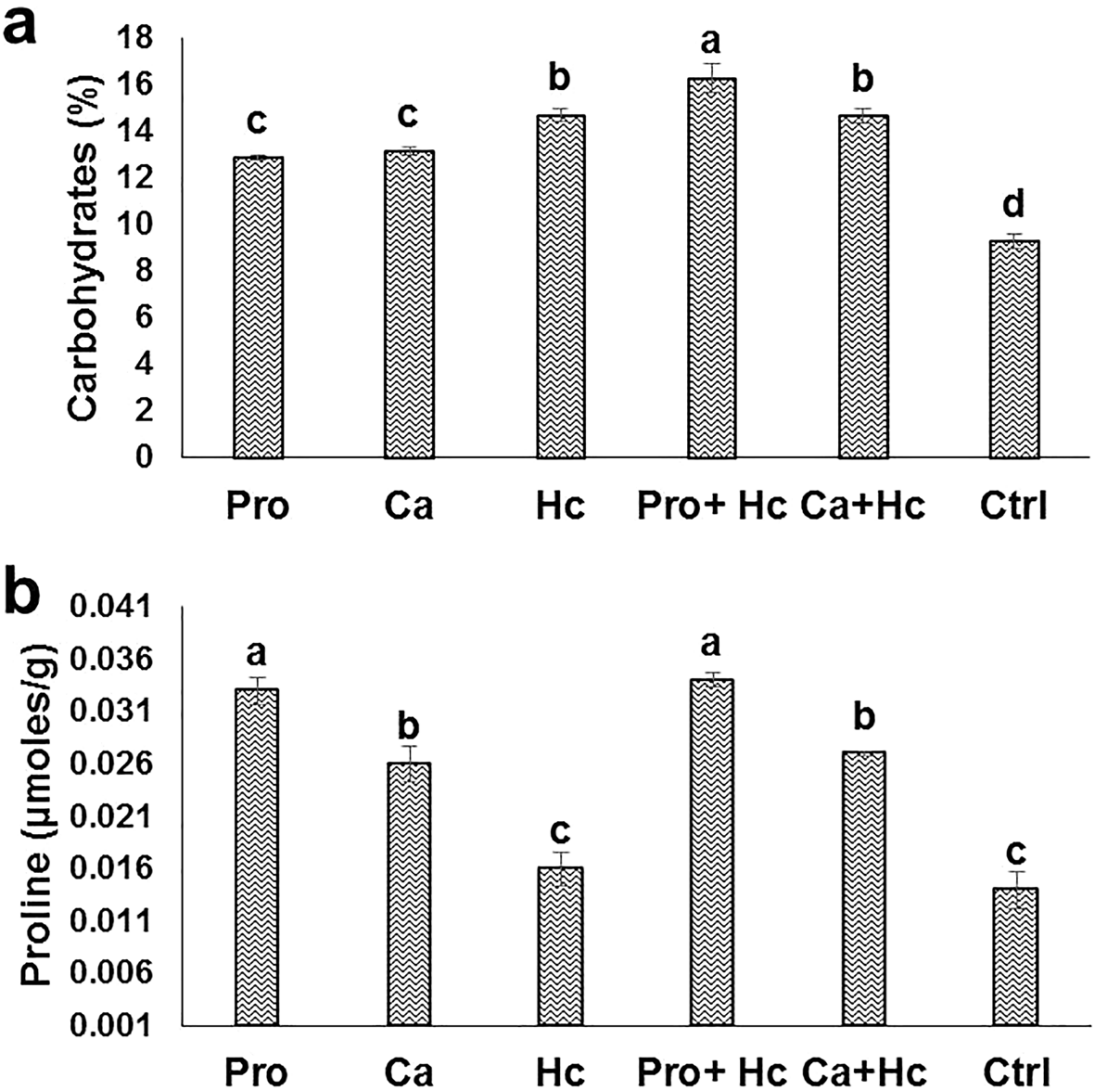
Effect of proline (Pro) and calcium (Ca) as foliar application alone or incombination with humic acid (Hc) as soil application on carbohydrates leaves percent (**a**) and proline (**b**) leaves content (µmoles g^−1^) of ‘Wonderful’ pomegranate trees grown under salt stress conditions. The different letters on the bars in each column represent significant difference at *p* ≤ 0.05; standard error bars represent standerd error of three replicates. Pro: 5 mM proline alone as foliar application; Ca: 1.5% calcium as foliar application alone; Hc: 15 g humic acid as soil application alone; Pro + Hc: 5 mM porline and 15 g humic acid; Ca + Hc: 1.5% calcium and 15 g humic; Ctrl: control (non treated).

Generally Hc treatment failed to increase proline leaves content compared with other conducted treatments (Fig. 1a). Treatments of Ca alone or with Hc increased leaves proline content by 85.7%, 92.85%, respectively compared to the control. For effects of foliar and soil application of different salt stress ameliorative materials (Fig. 1b), it can be observed that, Hc applied by soil succeeded in increasing carbohydrates percent by 58% compared to the control. Moreover, treatment of Pro alone or with Hc significantly increased the proline content of leaves by 135.71% and 142.85%, respectively compared to the control. Treatments of calcium alone or with Hc increased leaves proline content by 85.71% and 92.85% compared to the control.

### Nutritional status

**Figure 2:**
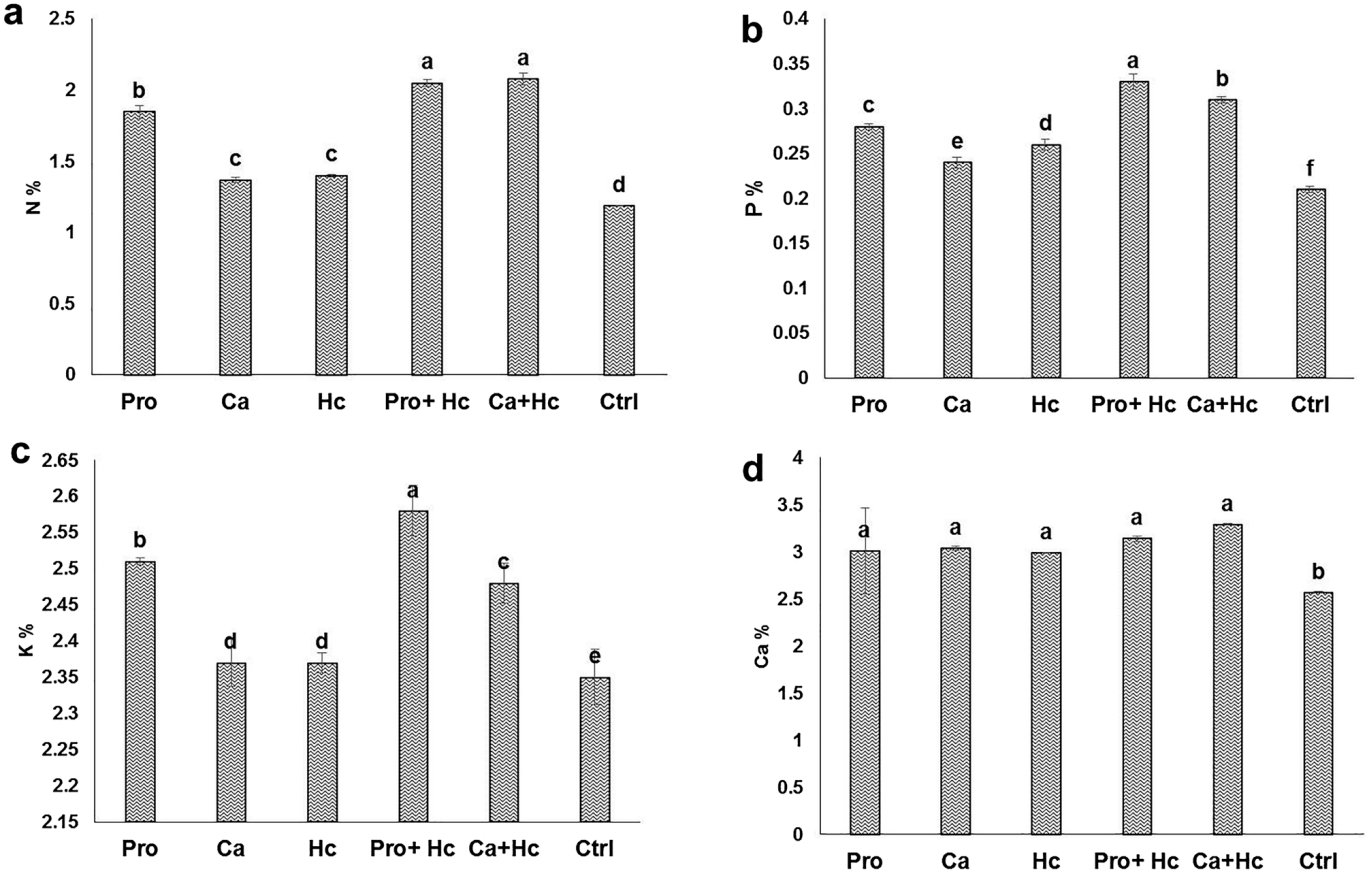
Effect of Proline (Pro) and Calcium (Ca) as foliar application alone or incombination with humic acid (Hc) as soil application on (**a**) nitrogen (N), (**b**) phosphorous (P), (**c**) potassium (K) and (**d**) calcium (Ca) leaves percent based on leaves dry weight of ‘Wonderful’ pomegranate trees grown under salt stress conditions. The different letters on the bars in each column represent significant difference at *p* ≤ 0.05; errors bars are standerd error of three replicates. Pro: 5 mM proline alone as foliar application; Ca: 1.5% calcium as foliar application alone; Hc: 15 g humic acid as soil application alone; Pro + Hc: 5 mM porline and 15 g humic acid; Ca + Hc: 1.5% calcium and 15 g humic; Ctrl: control (non treated).

Individual application of Hc succeeded in increasing the plant N, P, K, Ca concentrations significantly by 17.6%, 23.8%, 0.85%, 16.34%, respectively compared to the untreated control (Fig. 2a–d). Moreover, integrated application of Hc with Pro significantly increased leaves N, P, K and Ca by 72.26, 57.14%, 9.78% and 22.17%, respectively. In addition, co-application of humic acid with Ca significantly increased leaves N, P, K and Ca by 74.78%, 47.61%, 5.53% and 28%,and respectively.

### Toxic elements and inorganic osmolytes for osmoregulation

**Figure 3:**
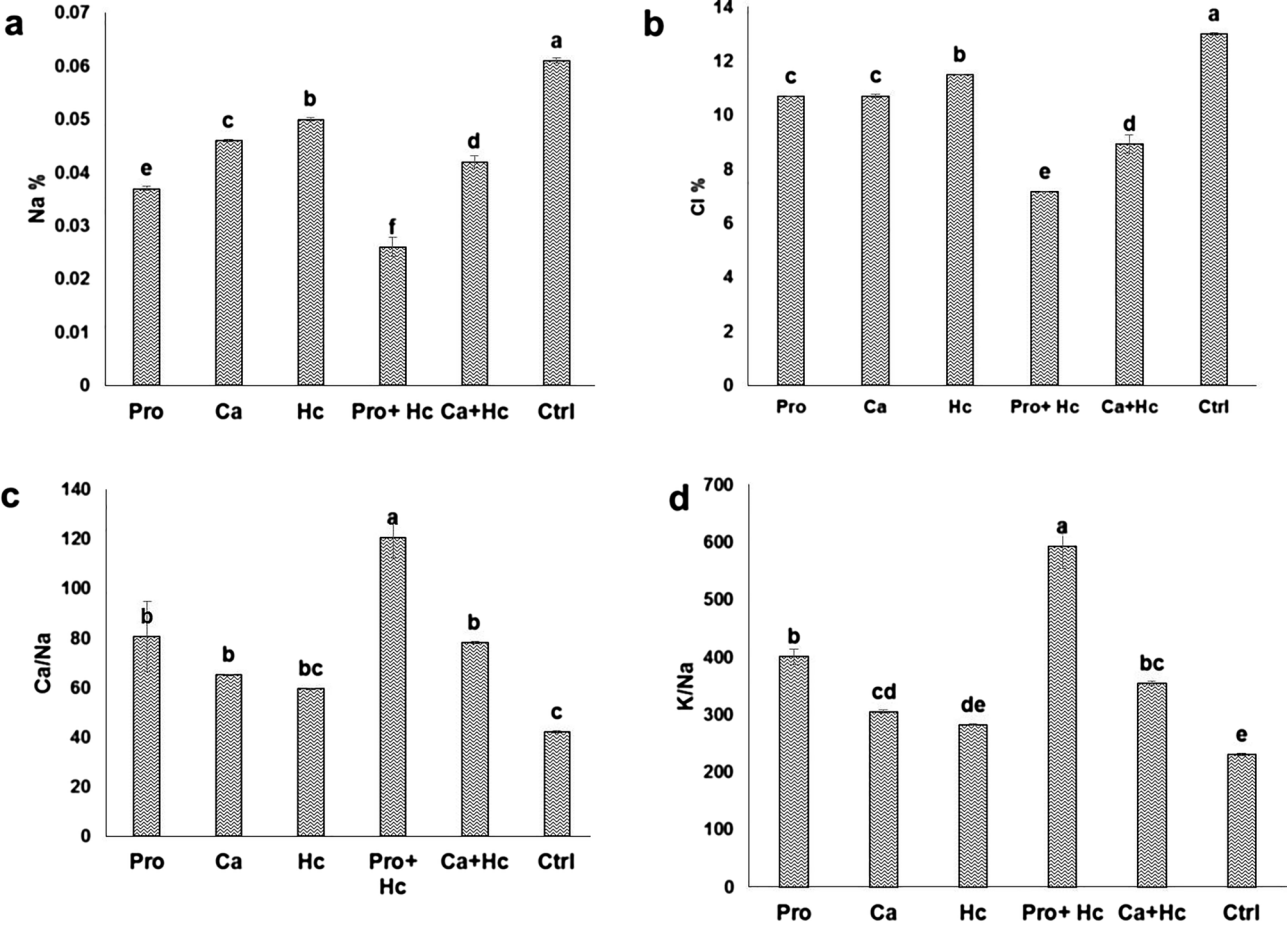
Effect of Proline (Pro) and Calcium (Ca) as foliar application alone or incombination with humic acid (Hc) as soil application on (**a**) leaves sodium (Na), (**b**) chloride (Cl), (**c**) calcium/sodium ratio (Ca/Na), (**d**) potassium/sodium ratio (K/Na) based on leaves dry weight of ‘Wonderful’ pomegranate trees grown under salt stress conditions. The different letters on the bars in each column represent significant difference at *p* ≤ 0.05; errors bars are standerd error of three replicates. Pro: 5 mM proline alone as foliar application; Ca: 1.5% calcium as foliar application alone; Hc: 15 g humic acid as soil application alone; Pro + Hc: 5 mM porline and 15 g humic acid; Ca + Hc: 1.5% calcium and 15 g humic; Ctrl: control (non treated).

Individual application of Hc or Pro or Ca significantly reduced the concentrations of the toxic elements; Na and Cl (Fig. 3a,b) compared to the control. The percent decrease in leaf Na was 94.59%, 64.86%, 51.35%, 40.54% and 29.72% for Pro+Hc, Pro alone, Ca+ Hc, Ca alone and Hc alone, respectively. Also, the decrease in Cl% recorded by 44.79%, 31.28%, 17.76%, 17.61% and 11.53% for Pro+Hc, Ca+Hc, Pro alone, Ca alone and Hc alone, respectively compared to the control. For the inorganic osmoregulation mechanisms (Fig. 3c,d). Hc acid alone failed to achieve a significant increase the concentration of osmoregulated solutes (Ca/Na ratio and K/Na ratio). While the integrated application of Hc plus Pro followed by Ca gave the highest values with significant values.

Regarding the effect of humic acid on plant nutritional status, our results indicated that, humic acid led to a significant increase in leaf N, P and K concentrations, while it led to a significant decrease in leaf Cl and Na concentration (Fig. 3). In this regard, humic acid has been associated with the preferential accumulation of these nutrients for several fruit trees such as Egyptian lime^37^, olives^38^, date palm^39^, grape^8^ and Water melon^14^. The beneficial effect of humic acid in improving the nutritional status of leaves under salt stress conditions may be due to their effect on increasing soil organic matter which subsequently increase soil holding capacity^11^, improve the exchange capacity, enhance mineral chelation^12^, increase available mineral, improve root growth^11^ and maintain nutrients from leaching out^37^. Most beneficial effect of humic acid under salt stress condition is the uptake limitation of toxic such as Na and Cl. These findings were previously reported in many fruit trees like Mexican lime^12^ and grape^8^. The results indicated a non significant effect of humic acid on leaf proline content, the application of humic acid decreased proline concentration in citrus^16^.

These results indicate that pomegranate plants have a strong capability to restrict Na and Cl accumulation in leaf tissues. In this regard, Sun et al.^4^ concluded that pomegranate plant is very tolerant to saline water irrigation up to an EC of 15 dS m^−1^ with little foliar salt damage and a slight growth reduction.

As for the effects of foliar Pro treatments under salt stress conditions, our results indicate that exogenous Pro significantly increased leaf K, Ca, proline contents, while it significantly decreased leaf Na and Cl concentrations. Most studies have been tested foliar spraying of proline on field crops and vegetable crops such as common beans^9,40^ mustard^41^, barley^42^, *Aloe vera*^43^, rice^44^, onion^20^ and pea plant^45^. Lima-Costa^46^ found that, exogenous proline at 5 mM improved vegetative growth of citrus plants under 100 mM NaCl. Proline application for *Simmondsia chinensis* at 20 mM significantly increased N, K and significantly decreased Cl and Na under salt stress conditions^47^. The results showed the great effect of Pro on improving plant nutritional status. Also, the integrative spraying of 6 mM Proline in addition to potassium silicate increased leaf N, P, K concentrations^9^ and decreased leaf Na concentration of common bean under NaCl at 150 mM^9,22^. Moreover, application of 10 mM proline increased leaf N, P, K and yield of sugar beet under drought stress^48^. In addition, inculated barley plants with two strains produced proline and IAA, enhancing plant water and nutrient uptake^42^. Exogenously application of proline of drought stressed onion improved photosynthetic efficiency, up-regulating osmoprotectants and water use efficiency^20^.

The results indicated that Ca led to a significant increase in leaf Ca, K, proline contents as well as significantly decreased leaf Cl and Na concentrations. These results were agreed with Ahmad et al.^49^ who treated tomato plants with Ca after salt exposure that reduced Na uptake. Also, Jasim et al.^50^ treated Berhi date palm with Ca which led to a significant increase in K^+^ and K/Na ratio in leaves, whereas Cl concentration was decreased. Moreover, Jasim et al.^50^ found that, Ca significantly increased leaf K^+^ and K^+^/Na^+^ ratios in date leaves, whereas Cl^−^ concentration were decreased. Ca is nesseccary for uptake K^+^ versus Na^+^^23–25^. Ca regulate some stress adaption mechanism such as cell polarity^51^, stomatal closure^52^, membrane stability and prevent the leakage of the solute from plant cell cytoplasm^53^. Ca plays an important role in cell elongation, cell division, membrane permeability, nitrogen metabolism and carbohydrate translocation^54^. In general, Ca is mostly applied to field crops and vegetable crops such as *Festuca ovina*^55^, endives^56^, tomato^49^, indica rice^57^, Legume^58^, wheat^59^ and pepper^60^. In ‘Elstar’ apple, leaf Ca concentration increased after Ca spraying at 6 to 9 kg ha^−1^^61^. On the other hand, Hagagg et al.^62^ found there was no clear effect of Ca treatments (3%, 5% and 7% CaCo_3_) on the mineral content of ‘Kalamata’ and ‘Manzanillo’ olive trees.

Under salt stress conditions, management of both root system (Hc treatment) and vegetative system (Ca, Pro treatments) increased K/Na ratio, Ca/Na ratio, carbohydrates and nutrients as well as decreased toxic elements (Na, Cl) suggests the mechanism of using pomegranate K, Ca and proline as osmoprotectants. Moreover, Pro treatments have achieved the most promising effects to alleviate salt stress may be come from providing plant energy, carbohydrates and nutrients from producing more endogenous proline content and becoming more nutritional balanced with lower toxic nutrients. Thus providing nitrogen to various roots, leaves and fruits building plant tissues. Furthermore, professional treatments have achieved the most promising effects of salt stress relief that come from providing plant energy to produce more endogenous proline content and become more balanced with lower toxic nutrients.

### Yield

**Figure 4:**
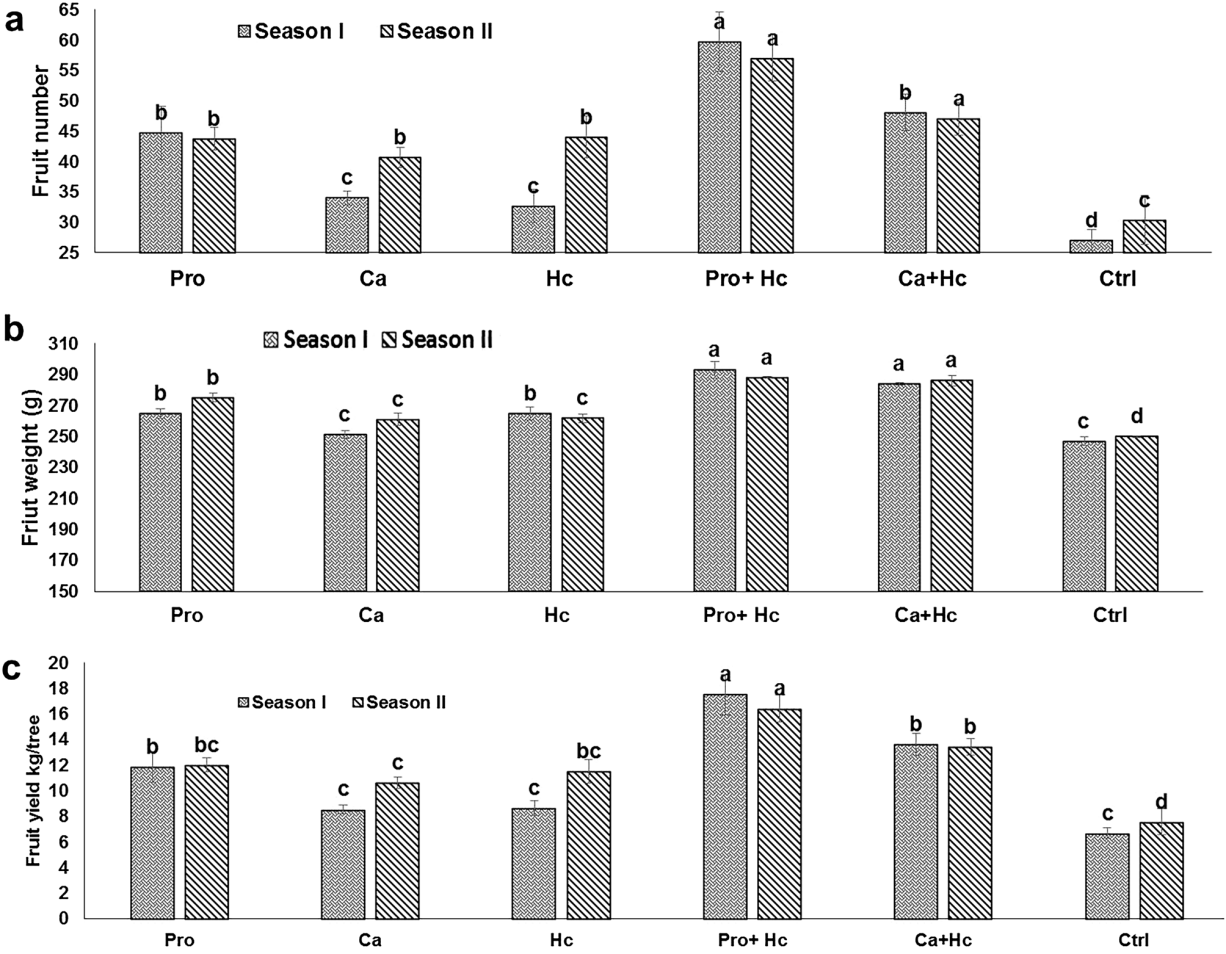
Effect of Proline (Pro) and Calcium (Ca) as foliar application alone or incombination with humic acid (Hc) as soil application on (**a**) fruit number, (**b**) fruit weight (g) and (**c**) yield (kg/tree) of ‘Wonderful’ pomegranate trees grown under salt stress conditions. The different letters on the bars in each column represent significant difference at *p* ≤ 0.05; errors bars are standerd error of three replicates. Pro: 5 mM proline alone as foliar application; Ca: 1.5% calcium as foliar application alone; Hc: 15 g humic acid as soil application alone; Pro + Hc: 5 mM porline and 15 g humic acid; Ca + Hc: 1.5% calcium and 15 g humic; Ctrl: control (non treated).

Single use of Hc resulted in a significant increase in fruit number, weight and yield compared to the untreated control (Fig. 4a,b,c). The percentage increase in fruit number due to Hc application was 45% and 21%, for fruit weight was 4.6% and 7.2% whereas for fruit yield were 29.39 and 51.84% for the first and second seasons, respectively. Also, the integrated application of Hc plus Pro increased fruit number by 121%, 87.93%, fruit weight by 15.2%, 18.7%, fruit yield by 162.6%, 116.4% for the first and second seasons, respectively. Furthermore, integrated application of Hc plus Ca increased fruit number by 54.96% and 77.77%, fruit weight by 14.98%, 14.4%, fruit yield by 77.17% and 104.28%, for the first and second season, respectively.

### Fruit quality

The combined use of Hc and Ca significantly increased fruit pulp/peel ratio followed by the application of Hc plus Pro (Fig. 4a,b,c). Both Hc acid or Pro alone failed to increase fruit TSS concentration, while during the first season the integrated application of Hc plus Ca increased fruit TSS significantly by 12.38% compared to the control. The integrated application of Hc plus Ca gave the highest fruit firmness in the first season with a significant value by 18.99% compared to the control.

Pro, Ca and Hc treatments increased nutrionnal status (N, P, K, Ca) as well as increasing carbohydates and proline as osmoprotectants mechanism which increased the supply of these growth-stimulated nutrients to different plant organ resulting in increased fruit yield.

**Figure 5:**
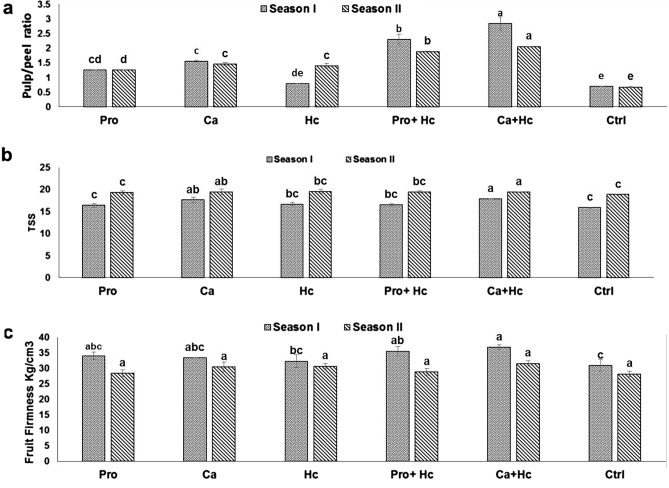
Effect of Proline (Pro) and Calcium (Ca) as foliar application alone or incombination with humic acid (Hc) as soil application on (**a**) fruit pulp/peel ratio, (**b**) fruit TSS percent, (**c**) fruit firmness (kg/cm^3^) (kg/tree) of ‘Wonderful’ pomegranate trees grown under salt stress conditions. The different letters on the bars in each column represent significant difference at *p* ≤ 0.05; errors bars are standerd error of three replicates. Pro: 5 mM proline alone as foliar application; Ca: 1.5% Calcium as foliar application alone; Hc: 15 g humic acid as soil application alone; Pro + Hc: 5 mM porline and 15 g humic acid; Ca + Hc: 1.5% calcium and 15 g humic; Ctrl: control (non treated).

With regard to fruit yield, the results indicated that humic acid has a great effect on increasing ‘Wonderful’ fruit weight and number subsequently fruit yield besides the pulp/peel ratio (Fig. 5). These results of the application of humic acid on increasing fruit yield were previously observed in many plants such as Egyptian lime trees^37^ and mango^63^. Recently, Masoud et al.^64^ found that, foliar application of ‘manfalouty’ pomegranate trees with 1% humic acid during the fruit growth improved fruit yield and quality. Increasing ‘Wonderful’ pomegranate yield under salt stress condition may be due to enhanced soil and plant mineral availablility and content. The main components of soil organic matter are humic substances (65–70%), which improve plant growth due to increased cell membrane permeability, phosphorus and oxygen uptake, physiological processes (photosynthesis, respiration) and root cell growth^65^. Finally, humic acid has direct effects on plant growth and nutritional status beside their great indirect effects on the soil^66^.

The results showed a role of Pro in improving yield and fruit quality. These results were in agreement with El Sayed et al.^67^ as they found that spraying ‘Manfalouty’ proline at 100 ppm improved fruit weight, TSS and yield. Also, application of 10 mM proline increased sugar beet yield under drought stress^48^. Increasing fruit number via proline application has been mentioned by Mattioli et al.^68^ as they reported that under salinity stress proline accumulated in pollen grains protected pollen fertility and improved yield stability.

The results showed that, Ca treatment significantly increased fruit weight, fruit number and pulp/peel ratio. These reults are in harmony with Masoud et al.^64^ who mentioned that, spraying ‘Manfalouty’ pomegranate trees with 2% calcium chloride significantly increased fruit yield. Increasing leaf Ca content of ‘Elstar’ apple after Ca spraying enhanced yield at 6–9 kg ha^−1^^61^. Calcium has been shown to mitigate the harmful effects of salinity on various plant species^49,55–60^.

The presence of Ca ions alleviated the toxic effects of salinity by promoting tissue growth. These effects may be resulting from the role of Ca in plant cell elongation and division, permeability of cell membrane, nitrogen metabolism and carbohydrate transport^54^. In fruit trees, spraying ‘Samany’ and ‘Zaghloul’ date palm with 5% calcium carbonate three times increased bunch weight and consequently total yield, fruit weight, thickness and TSS content^69^. Similar results were found in pomegranate^64^, olive^62^ and date palm^69^.

## Conclusion

Individual application of humic acid (15 g/tree) as soil application or proline (5 mM) and Ca (1.5%) as foliar application three times a season was successful in alleviating the adverse effect of salt stress in ‘Wonderful’ pomegranate trees. The integrated applications of humic acid in addition to foliar application of proline or calcium counteract the negative effect of salinity via increasing organic and inorganic organic osmolytes, improving plant nutritional status and reducing the uptake of toxic elements which resulted in increasing fruit yield.

## Data Availability

The data generated and/or analysed during the current study are available per request to the corresponding author.
